# Allergen-Specific Immunotherapy Alters the Frequency, as well as the FcR and CLR Expression Profiles of Human Dendritic Cell Subsets

**DOI:** 10.1371/journal.pone.0148838

**Published:** 2016-02-10

**Authors:** Kristina Lundberg, Frida Rydnert, Sissela Broos, Morgan Andersson, Lennart Greiff, Malin Lindstedt

**Affiliations:** 1 Department of Immunotechnology, Lund University, Lund, Sweden; 2 Department of Otorhinolaryngology, Head & Neck Surgery, Skåne University Hospital, Lund, Sweden; University of Bergen, NORWAY

## Abstract

Allergen-specific immunotherapy (AIT) induces tolerance and shifts the Th2 response towards a regulatory T-cell profile. The underlying mechanisms are not fully understood, but dendritic cells (DC) play a vital role as key regulators of T-cell responses. DCs interact with allergens via Fc receptors (FcRs) and via certain C-type lectin receptors (CLRs), including CD209/DC-SIGN, CD206/MR and Dectin-2/CLEC6A. In this study, the effect of AIT on the frequencies as well as the FcR and CLR expression profiles of human DC subsets was assessed. PBMC was isolated from peripheral blood from seven allergic donors before and after 8 weeks and 1 year of subcutaneous AIT, as well as from six non-allergic individuals. Cells were stained with antibodies against DC subset-specific markers and a panel of FcRs and CLRs and analyzed by flow cytometry. After 1 year of AIT, the frequency of CD123^+^ DCs was increased and a larger proportion expressed FcεRI. Furthermore, the expression of CD206 and Dectin-2 was reduced on CD141^+^ DCs after 1 year of treatment and CD206 as well as Dectin-1 was additionally down regulated in CD1c^+^ DCs. Interestingly, levels of DNGR1/CLEC9A on CD141^+^ DCs were increased by AIT, reaching levels similar to cells isolated from non-allergic controls. The modifications in phenotype and occurrence of specific DC subsets observed during AIT suggest an altered capacity of DC subsets to interact with allergens, which can be part of the mechanisms by which AIT induces allergen tolerance.

## Introduction

Allergen-specific immunotherapy (AIT) is the only cure for allergic diseases. On repeated administration of hyposensitizing allergens, the immune reaction shifts from a Th2 profile towards a tolerogenic response. As reviewed by Akdis *et al*. [1], effects induced by AIT include e.g. generation of regulatory T-cells and B-cells as well as increased levels of allergen-specific IgG4 [1,2]. However, the full underlying mechanisms responsible for immune cell alterations associated with AIT remain elusive.

Dendritic cells (DCs) are professional antigen presenting cells that determine the activity and nature of T-cell responses [3]. Accordingly, DCs play an important role for shifting the T-cell response towards tolerance during the course of AIT. In a study using DCs obtained from patients receiving rush protocol AIT for venom hypersensitivity, the number of blood CD123^+^ DCs (plasmacytoid DCs, pDCs) decreased 52 hours after AIT initiation, whereas the levels of CD11c^+^ DCs (myeloid DCs, mDCs) remained unaltered [4]. However, the CD11c^+^ DC population is comprised of several subsets, each identified by their unique expression of CD1c, CD141 and CD16 and subset-specific functionality is evident [5,6]. Thus far, the effect of AIT on the frequencies and phenotype of these distinct subsets has not been determined.

Fc receptors are involved in the recognition of allergens by DCs via immunoglobulin (Ig) binding and thereby influence allergen-specific T-cell responses [7,8]. For example, FcεRI-mediated uptake of grass pollen allergen by monocyte-derived dendritic cells (MoDCs) augments Th2 responses [8] and cat allergen targeted to CD64 (FcγRI) on MoDCs produce an altered Th2 response with regulatory features [7]. Interestingly, 1 year of AIT has been reported to reduce the expression of CD32/Fcγ receptor II (FcγRII) on CD11c^+^ mDCs [4]. However, expression levels were not determined on specific subsets of mDCs and it is not known if expressions of other FcRs are altered on specific DC subsets during the course of AIT.

In addition to FcRs, CLRs are associated with allergic responses [9–14]. Although generally known as pattern-recognition receptors involved in pathogen recognition [15], it has recently become clear that CLRs including Dendritic cell-associated C-type lectin 2 (Dectin-2), CD206/Macrophage Mannose receptor (MR) and CD209/Dendritic Cell-Specific Intercellular adhesion molecule-3-Grabbing Non-integrin (DC-SIGN) can interact with various allergens and mediate Th2 cell activation by DCs [9–13]. Furthermore, studies in mice indicate that Dectin-1 has an immunopathogenic role in allergic responses to the fungi *Aspergillus fumigatus* [14] and human mDCs activated via Dectin-1 are shown to decrease Th2 responses, whereas the opposite effect was shown for pDCs [16]. Another reason why CLRs are relevant in the context of allergic diseases is that targeting of CLRs have been shown to induce tolerance. It is for instance well established that targeting antigen to CD205/DEC205 without an adjuvant induces tolerance [17]. More recently, targeting antigen to CD301/C-type lectin superfamily member 14 (CLECSF14) on human DCs in vitro was shown to generate antigen-specific IL-10–producing CD4^+^ T cell responses and similar results were obtained *in vivo* using non-human primates [18]. Furthermore, mice studies by Joffre et al. suggests that antigens delivered to DNGR1/ C-type lectin domain family 9A (CLEC9A) in absence of an adjuvant leads to a regulatory T-cells response [19]. Thus, there is data supporting a role for DNGR1, CD301 and CD205 in tolerance induction and these could be potential targets for treatment of detrimental responses towards allergens. It is currently unknown if CLR expressions are altered on specific DC subsets during the course of AIT. Monitoring expression of CLRs before and during AIT can provide insights into their association with allergic immune responses and/or induction of tolerance and may reveal new targets for immunomodulation.

In a previous study, we demonstrated that DNGR1, which is uniquely expressed by CD141^+^ DCs, is displayed at lower levels on cells from allergic as compared to non-allergic individuals [20]. Primarily, DNGR1 is known for its ability to mediate cross-presentation and activation of CD8^+^ T-cells [21]. Interestingly, murine studies have shown that CD8^+^ T-cells are critical for the suppressive phenotype of regulatory T-cells in allergic airway disease [22]. Furthermore, as mentioned above, antigens targeted to DNGR1 are shown to induce tolerance in mice [19]. Hence, the reduced expression of DNGR1 on CD141^+^ DCs from allergic individuals raises the question of whether DNGR1 is involved in tolerance induction towards allergen. It is presently not known if DNGR1 expression on CD141^+^ DCs is affected by AIT.

In this study, we examined the frequencies of DC subsets as well as their expression of different FcRs and CLRs before treatment and after 8 weeks and 1 year of AIT. We demonstrate that the frequency of CD123^+^ DCs, as well as their expression of FcεRI, was increased by AIT. Furthermore, expression of CD206 and Dectin-2, which are known to interact with allergens, was reduced on CD141^+^ DCs after 1 year of treatment, and CD206 as well as Dectin-1 were additionally down regulated by CD1c^+^ DCs. By contrast, an increase in DNGR1 expression on CD141^+^ DCs was demonstrated during AIT, reaching levels similar to those of cells from non-allergic individuals. Collectively, the presented data suggest that AIT modifies the capacity and routes by which specific DC subsets interact with allergens.

## Method

### Subjects and blood sampling

Seven allergic patients undergoing AIT, as well as six non-allergic individuals, participated in the study. Before the study, AIT subjects were evaluated by clinicians and qualified for the treatment based on disease history, levels of allergen-specific IgE, the result of skin prick tests and displayed severe allergic symptoms that were not relieved by standard medication and/or strategies to limit allergen exposure. Donor sensitizations, as assessed by skin prick tests and allergens used for the treatment are outlined in Table 1. AIT was performed according to a standard clinical protocol with increasing doses of allergens administered subcutaneously. Blood was sampled, using heparin as anti-coagulant, at three time-points: immediately before the initiation of AIT (t0), when a dose of 10.000 SQ units (Alutard, ALK-Abelló A/S, Hørsholm, Denmark) was reached after approximately 8 weeks (t8w) and after 1 year of AIT when the maximum allergen dose (100.000 SQ units) had been administrated for about six months (t1y). The immunotherapy was started in the autumn outside the pollen season. Hence, blood was drawn at t0, t8w and t1y when patients were not exposed to ambient pollen allergens and did not medicate. Notably, t8w and t1y were scheduled approximately 1 week after last allergen administration. Serum levels of IgG4 and IgE specific for the allergens used for AIT were monitored with Immunocap (Thermo Scientific, Allerød, Denmark) at the three time points (serum from donor 6 was obtained at t8w and t1y only). In line with the literature [1,2], levels of IgG4 were drastically increased during the course of AIT (S1 Fig). Furthermore, in accordance with previous studies, IgE levels were initially increased from t0 to t8w followed by a decline from t8w to t1y for the great majority of assessments. Non-allergic controls were similarly sampled outside pollen seasons (around t8w). It should be noted that the ages of non-allergic donors were slightly higher than the ages of allergic subjects. Nonetheless, all subjects were adults and none were elderly. Furthermore, apart from DNGR1, expression of the investigated CLRs by DCs from non-allergic donors was shown to be similar to DCs from allergic donors before AIT, according to a previous study by us [20]. Apart from the allergy diagnosis, all donors were considered healthy. The study was approved by the regional Ethics Committee in Lund (Sweden) and written informed consent was obtained from all donors.

**Table 1:** Donor characteristics and allergens used for AIT.

Don	Sensitizations (Skin prick test)	Allergens for AIT	Age and gender
1	BP, GP, CD, DD	3-tree, 5-grass, *Fel d*	M, 30
2	BP, GP	3-tree, 5-grass	M, 30
3	BP, GP, HDM, CD, DD	3-tree, 5-grass, *Der p*	F, 36
4	HDM, CD, DD, MO	*Der p*	M, 23
5	BP, MP, HDM, MO	3-tree, *Art v*, *Der p*	F, 41
6	BP, GP, MP	3-tree, 5-grass, *Art v*	M, 27
7	BP, GP, CD, DD, HD	3-tree, 5-grass, *Fel d*	F, 24
NA1	-	-	M,30
NA2	-	-	F, 42
NA3	-	-	F, 41
NA4	-	-	M, 39
NA5	-	-	F, 51
NA6	-	-	M, 35

Abbreviations: BP-birch pollen; CD-cat dander; DD-dog dander; DP- *Dermatophagoides pteronyssinus*; F-female; GP^1^-grass pollen; HD-horse dander; M-male; MO-mold; MP-mugwort pollen; NA-non-allergic.

Allergens for AIT: 3-tree: tree pollen mixture (alder, birch, hazel), 5-grass: grass pollen mixture (meadow foxtail, cocksfoot, meadow fescue, english ryegrass, timothy), Art v: Mugwort (*Artemisia vulgaris*), Der p: house dust mite (*Dermatophagoides pteronyssinus*), Fel d: cat dander (*Felis domesticus*).

### Sample preparation and antibodies

Immediately after blood sampling, peripheral blood mononuclear cells (PBMC) were isolated by density gradient centrifugation using Lymphoprep (Medinor, Lidingö, Sweden). After washing twice, antibody staining was performed as outlined previously (i.e. staining performed on fresh material) [20]. Antibodies used included rabbit anti-mouse-PE, CD14-PE, CD19-PE, CD19-FITC (Dako Cytomation, Glostrup, Denmark), goat anti-rabbit-FITC, CD123-biotin, CD123-PE, CD16-PeCy7, CD3-PE, CD3-FITC, CD32-FITC, SA-APCCy7 (BD Biosciences, San Jose, CA), CD1c-PE, CD141-APC, CD141-biotin (Miltenyi Biotec, Bergisch Gladbach, Germany), HLA-DR-PerCp-Cy5.5 (Biolegend, San Diego, CA), CD14-FITC, CD3-APC, CD14-APC and CD19-APC (Invitrogen, Carlsbad, CA), CD301-Alexa fluor 488 (Dendritics, Lyon, France), CD206-PE, CD207/Langerin-PE (Beckman Coulter, Brea, CA), CD205-FITC, FcεRI-biotin (eBioscience, San Diego, CA), Dectin-1/CLEC7A-FITC (AbD Serotec, Kidlington, UK), Dectin-2, DCIR/CLECSF6, DNGR1, CD64-PE (R&D Systems, Minneapolis, MN) and CD280/C-type mannose receptor 2 (MRC2), CD209 (Abcam, Cambridge, UK). Stringent staining procedures were followed to ensure robust performance over time. For example, after each staining step, samples were washed, centrifuged and resuspended to an exact volume to ensure equal dilution of the antibody. Also, all samples were blocked before staining with conjugated antibodies. Antibodies were used within their expiry date and equal performance of new lots was confirmed before replacement of antibodies that expired within one year. Furthermore, in accordance with instrument guidelines, the BD FACS Canto (BD Bioscience) flow cytometer used for analysis was checked daily using BD Cytometer Setup & Tracking beads (BD Bioscience) and adjustments were made using application settings to ensure equal performance at every analysis.

### Analysis

**Fig 1:**
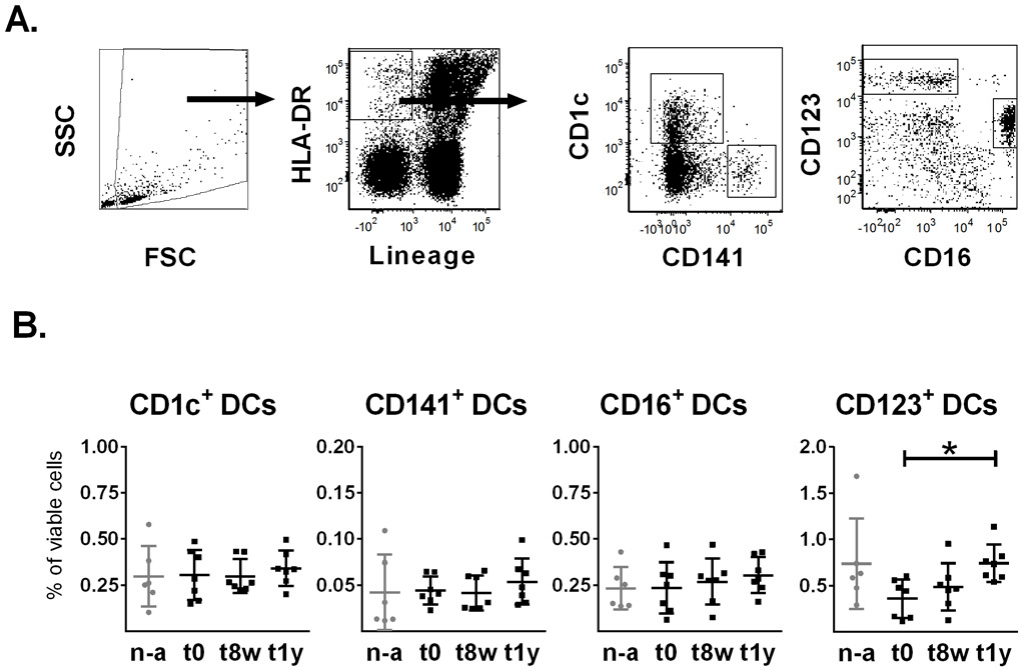
Gating of DC subsets and frequency of peripheral blood DCs. (A). Specific DC subsets were identified as HLA-DR positive, lineage negative cells that expressed either CD141, CD1c, CD16 or CD123, using flow cytometric analysis of viable cells in the FSC-SSC gate. (B). Flow cytometric analysis was performed on non-allergic controls (n-a) (n = 6) as well as on AIT subjects (n = 7) before treatment (t0), as well as after 8 weeks (t8w) and 1 year (t1y), to determine the frequencies of CD141^+^, CD1c^+^, CD123^+^ and CD16^+^ DC subsets. Plots show each donor value as well as mean values and SD variations. * = p<0.05, Friedman’s test followed by a Dunn's multiple comparison test.

The expression analysis was carried out as described elsewhere [20]. Briefly, FCS express (De Novo Software, Los Angeles, CA) was used for expression analysis and DC subsets were identified as lineage (CD3, CD14, CD19) negative and HLA-DR positive cells that additionally expressed one out of the four subset-selective markers CD1c, CD141, CD16 or CD123 (Fig 1A). In line with guidelines for accurate interpretation of multicolor flow cytometry data [23], the percentage of positive cells for each marker (PRR or Fc receptors) was assessed as compared to a Fluorochrome Minus One control (i.e. sample stained identically apart from the CLR or FcR antibody which was omitted in the control) and net values were calculated (representative gating of positive cells can be viewed in S2 Fig). Statistical calculations were performed in GraphPad Prism software (GraphPad Software, La Jolla, CA) and comparisons over time were made with a Friedman’s test followed by a Dunn's multiple comparison test. The statistical analysis of cells from AIT donors as compared to non-allergic donors was calculated using a Kruskal-Wallis test followed by a Dunn's multiple comparison test. Six non-allergic controls were analyzed and generally, cells from seven AIT patients were analyzed for expression of FcRs and CLRs on respective DC subsets (S1 Table).

## Results

### Peripheral blood CD123^+^ DCs frequencies increase after 1 year of AIT

The frequency of DC subsets, gated as outlined in Fig 1A, was investigated before the initiation of AIT (t0) and after 8 weeks (t8w) as well as after 1 year (t1y). At the latter time point, the CD123^+^ DC frequency showed a statistically significant increase as compared to before treatment (Fig 1B). Furthermore, the absolute number of CD123^+^ DCs was similarly increased at t1y, although this was not statistically significant (p = 0.0651). In contrast, the levels of CD1c^+^ DCs, CD141^+^ DC and CD16^+^ DCs were unaltered during the course of AIT. No differences were seen for AIT donors as compared to non-allergic controls.

### AIT alters expression of FcγRs and FcεRI on CD123^+^ and CD141^+^ DC subsets

**Fig 2:**
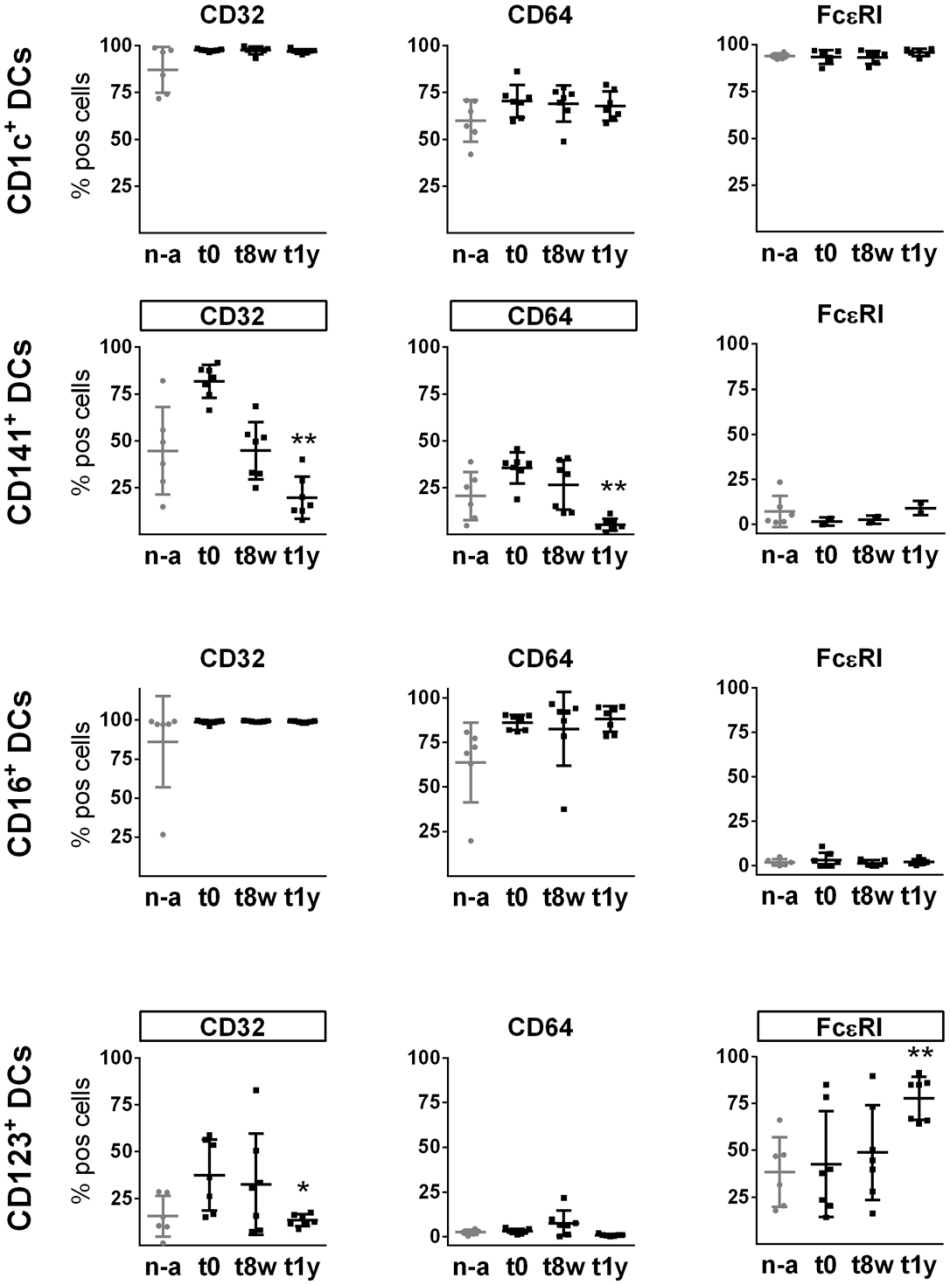
FcR expression on CD1c^+^, CD141^+^, CD123^+^ and CD16^+^ DC subsets from non-allergic controls as well as from AIT donors before and during the course of treatment. FcR expression based on flow cytometric analysis on cells from non-allergic controls (n-a) (n = 6) and AIT donors at t0, t8w and t1y (n = 7, with the exception of FcεRI on CD141^+^ DCs (n = 2) and CD1c^+^ DCs (n = 6)). Plots show each donor value as well as mean values and SD variations. * = p<0.05; ** = p<0.01, (Friedman’s test followed by a Dunn's multiple comparison test. FcRs altered significantly during AIT are framed. FcεRI on CD141^+^ DCs not tested for significance, too few AIT samples).

CD32 (FcγRII), CD64 (FcγRI) and FcεRI expression was analyzed on specific DC subsets before initiation of AIT, after 8 weeks and after 1 year of AIT (Fig 2) (representative gating of positive cells can be viewed in S2 Fig) and expression of FcRs was furthermore monitored on cells from non-allergic controls. None of the FcRs were significantly different for non-treated AIT donors as compared to non-allergic controls (Fig 2); however, the average expression of CD64 by CD16^+^ DCs was lower on cells from non-allergic controls than from allergic donors before treatment and the difference was close to significant (p = 0.0589). It should be noted however, that one donor had a lower expression of CD64 as well as CD32 on CD16^+^ DCs as compared to other donors, and the reason for this remained unknown. The other subsets from this donor displayed a comparable expression profile to the other donors. Furthermore, there was a trend that the frequency of CD141^+^ DCs as well as of CD123^+^ DCs expressing CD32 was lower for non-allergic controls as compared to AIT subjects at t0 (p = 0.0605 and p = 0.0769, respectively). Interestingly, after 1 year of AIT, CD64 and CD32 expression was significantly decreased on CD141^+^ DCs and expression of CD32 was additionally reduced on CD123^+^ DCs. Expression of CD32 on CD141^+^ DCs showed a decreasing trend already after 8 weeks, but this change did not reach statistical significance (p = 0.0651; S3 Fig). Expression of FcεRI increased on CD123^+^ DC after 1 year of treatment, whereas FcεRI was unaltered on CD1c^+^ and CD16^+^ DCs. Variation in the expression of FcεRI on CD141^+^ DCs over time could not be estimated due to a limited number of donors (n = 2), as a consequence of a temporary technical problem. Evident AIT-induced regulation of CD32, CD64 and FcεRI, as observed on CD123^+^ and CD141^+^ DCs, was not detectable on CD1c^+^ or CD16^+^ DCs.

### Marked changes in CLR profiles of CD123^+^, CD141^+^ and CD1c^+^ DCs, but not CD16^+^ DCs as a consequence of AIT

**Fig 3:**
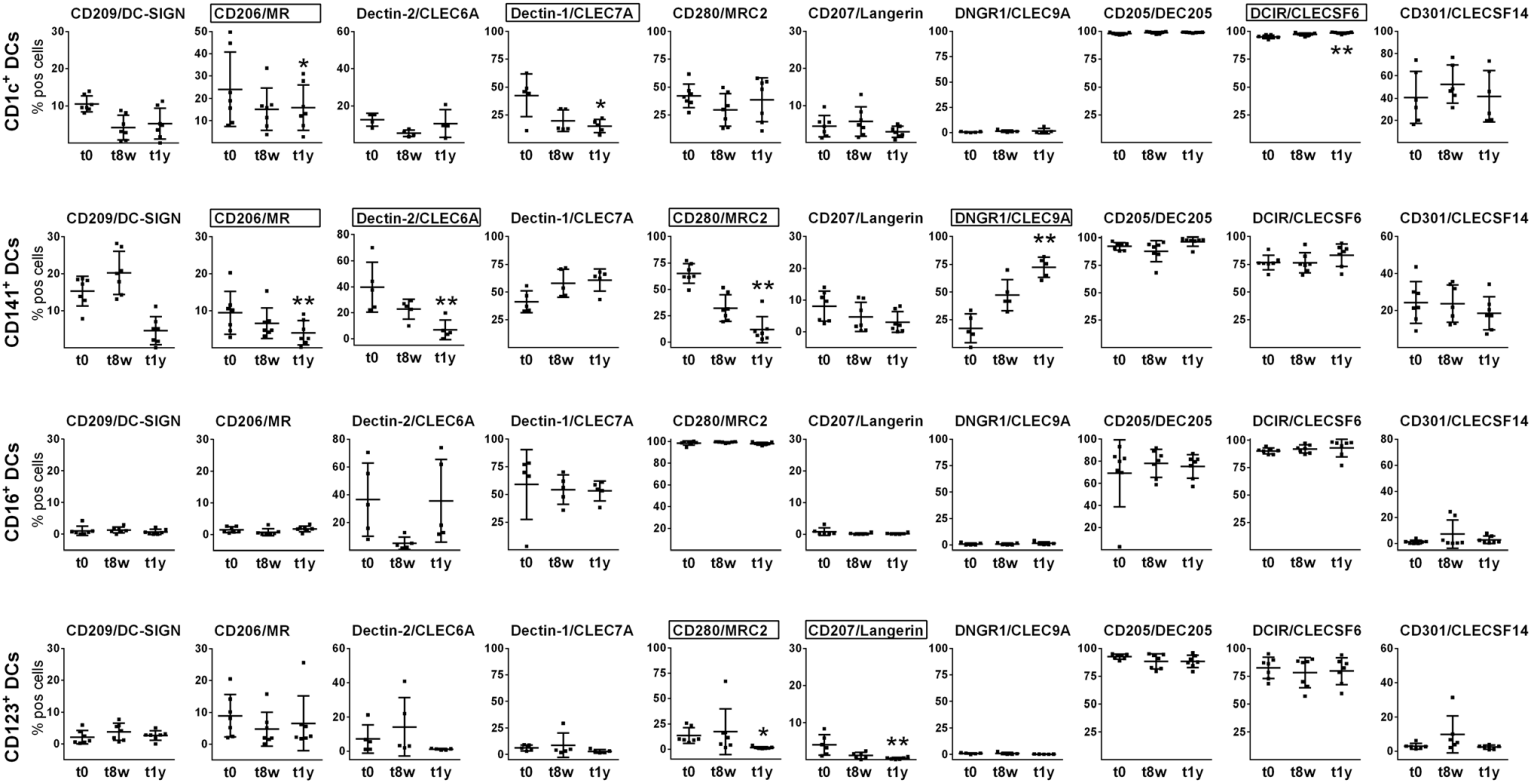
CLR profiles of CD1c^+^, CD141^+^, CD123^+^ and CD16^+^ DC subsets before and during the course of AIT. Phenotypic changes were assessed by flow cytometry at t0, t8w and t1y. Plots show each donor value as well as mean values and SD variations. Statistical significance was calculated by a Friedman’s test followed by a Dunn's multiple comparison test and significantly altered CLRs are framed (generally n = 7, see S1 Table). * = p<0.05; ** = p<0.01.

CLR expression profiles on CD123^+^, CD141^+^ and CD1c^+^ DCs were altered during AIT as compared to before treatment. As shown in Fig 3, expression of four, three and two CLRs were altered during the course of AIT on CD141^+^, CD1c^+^ DCs and CD123^+^, respectively. In contrast, the expression of CLRs on CD16^+^ DCs was unaffected.

Significantly reduced expression after 1 year of AIT was demonstrated for 1) CD206 on CD141^+^ and CD1c^+^ DCs, 2) Dectin-1 on CD1c^+^ DCs, 3) Dectin-2 on CD141^+^ DCs, 4) CD280 on CD141^+^ and CD123^+^ DCs, and 5) CD207 on CD123^+^ DCs. The most drastic reductions in mean percentage positive cells were seen for Dectin-1 on CD1c^+^ DCs as well as for Dectin-2 and CD280 on CD141^+^ DCs. In addition, expression of CD209 on CD141^+^ DCs decreased for all donors at t1y as compared to before treatment, although statistical significance was not reached (p = 0.0651, S4 Fig). Increased expression was detected for DNGR1 on CD141^+^ DCs and also for DCIR on CD1c^+^ DCs, although it was a minor increase of the latter. In contrast, the average frequency of CD141^+^ DCs expressing DNGR1 was increased >4-fold after 1 year of AIT. Overall, the CLR expression alterations are substantial considering the limited sample cohort, suggesting that these CLRs are strongly affected during AIT.

### DNGR1 expression by CD141^+^ DCs is restored by AIT

**Fig 4:**
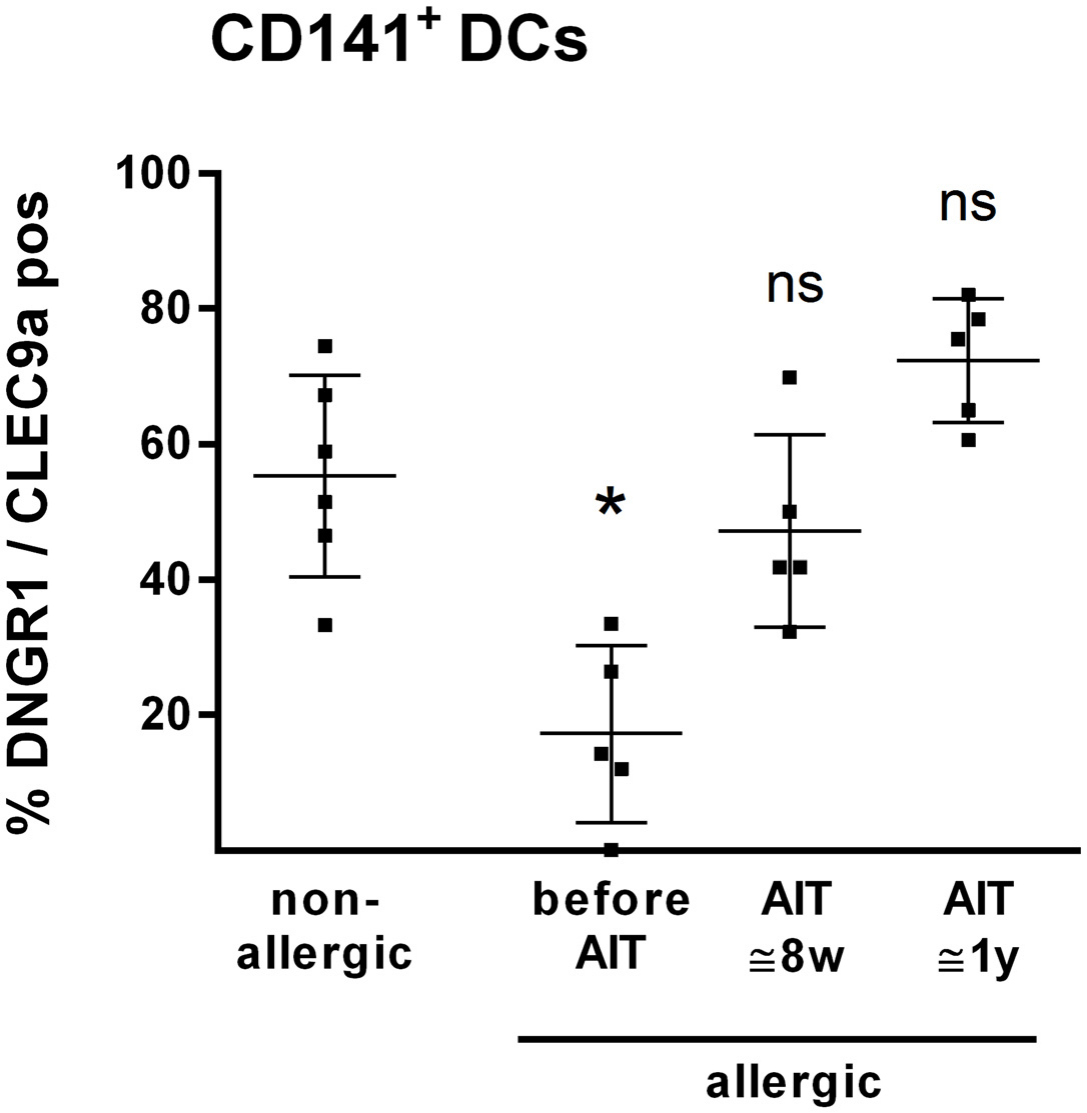
DNGR1 expression by CD141^+^ DCs was restored during AIT to levels comparable to non-allergic individuals. Flow cytometric analysis was performed on CD141^+^ DCs from non-allergic donors (n = 6), as well as allergic donors (n = 5) at t0, t8w and t1y. Plots show each donor value as well as mean values and SD variations. Statistical analysis of AIT donors compared to non-allergic donors was performed with a Kruskal-Wallis test followed by a Dunn's multiple comparison test. * = p<0.05. ns-non-significant; w-weeks; y-years.

DNGR1 expression is lower on CD141^+^ DCs from allergic as compared to non-allergic donors [20] and therefore DNGR1 expression determined during the course of AIT was compared to expression on cells from non-allergic individuals. The significantly increased DNGR1 expression on CD141^+^ DCs after 1 year of AIT (Fig 3), as compared to before treatment, resulted in a frequency of DNGR1 positive cells that was similar to that of cells from non-allergic individuals (Fig 4). Thus, DNGR1 expression on CD141^+^ DCs was restored during AIT.

## Discussion

In this study, we have shown AIT-associated modifications of the frequencies and receptor expression profiles of distinct human DC subsets in peripheral blood. While only the CD123^+^ DC subset frequency was affected after 1 year of treatment, expression of FcRs and CLRs was altered on several DC subsets. Interestingly, expression of CD206 and Dectin-2 that are previously demonstrated to bind allergen components, were significantly reduced on CD1c^+^ and/or CD141^+^ DCs after 1 year of AIT. Furthermore, the frequency of CD141^+^ DCs expressing DNGR1, which is reduced in allergic rhinitis patient samples [20], was significantly increased upon AIT and reached levels similar to those of cells from non-allergic donors. Taken together, these modifications suggest an altered capacity of DC subsets to interact with allergens during AIT, which may represent cellular events linked to the protective effect of the treatment.

An increase in the frequency of CD123^+^ DCs was observed after 1 year of AIT in this study, whereas the frequencies of CD1c^+^, CD16^+^ and CD141^+^ DCs were unaltered. (Note, the samples were obtained 1 week after the latest allergen administration.) It has previously been shown that allergen inhaled by patients with allergic asthma leads to decreased numbers of circulating CD11c^+^ mDCs already after 24 hours [24]. By contrast, analysis of cellular frequencies 1 week after allergen challenge of individuals with allergic rhinitis did not result in detectable changes of blood DC subsets [25]. Additionally, it was reported that blood DC subsets were unaltered 24 hours after six weekly skin allergen provocations, while decreased frequencies of blood CD1c^+^ DCs as well as CD123^+^ DC were observed 24 hours after the first allergen injection [26]. Thus, DC subset levels appear to be affected transiently by allergen exposure and then balanced out within a week as well as after repeated exposure, possibly due to stimulated DC differentiation in the bone marrow. Although no alterations were seen in the present study after 8 weeks of AIT, the significantly up regulated frequency of CD123^+^ DCs demonstrated after 1 year suggests that a new equilibrium had been reached at that time point. In contrast to our observation, blood pDC numbers have previously been shown to be unaltered after 1 year of rush venom immunotherapy [4]. The discrepancy may be due to the antigen and/or that rush protocol uses faster dose escalations as compared to the standard protocol used in the present study and cell equilibriums may thus be reached with different kinetics. Interestingly, evidence supporting a tolerogenic function of pDCs is emerging. For example, pDCs are shown to have a protective role against Th2 inflammation in mice [27,28] and human CD123^+^ pDCs are superior to mDCs at inducing regulatory T-cells [29]. Furthermore, we recently demonstrated that human CD123^+^ pDCs, in contrast to CD1c^+^ mDCs, were unable to activate allergen-specific Th2 cells [6]. The increased CD123^+^ pDC levels shown after 1 year of AIT in this study could thus be an explanation for the well-documented induction of regulatory T-cells upon AIT [1] and part of the mechanism behind therapy efficacy.

The altered levels of FcRs on CD123^+^ and CD141^+^ DCs observed during the course of AIT in this study suggest an adjustment of Ig-mediated allergen internalization. Reduced expression of CD32 was found on CD123^+^ DCs after 1 year of AIT, and both CD64 and CD32 were decreased on CD141^+^ DCs, which is in line with reports demonstrating reduced CD32 expression on mDCs after 1 year of venom AIT [4]. IgG receptor expression are likely to influence immune responses induced by CD141^+^ DCs as cross-presentation of antigen by these cells is facilitated by antigen-specific IgGs [30]. Also, CD32 mediates antigen presentation by CD123^+^ DCs [31]. Thus, the down regulated FcγR (CD32/CD64) expression by CD141^+^ DCs and CD123^+^ DCs during AIT suggests that IgG-mediated antigen presentation by these subsets is reduced. The ultimate outcome of altered FcRs on DC subsets will also be affected by the presence of allergen-specific antibodies at different time points and IgG4-mediated allergen responses may thus be more pronounced upon AIT as compared to before treatment (increased levels of allergen-specific IgG4 upon AIT, see S1 Fig). Among the FcRs, IgG4 has highest affinity for CD64 [32] and the increased levels of IgG4 might thus primarily affect DCs other than the CD141^+^ DCs as this subset showed down regulated CD64 expression upon AIT. In addition to IgG receptors, CD123^+^ DCs displayed increased expression of FcεRI after 1 year of AIT. Allergen uptake via FcεRI on MoDCs stimulates Th2 responses [8], but whether or not FcεRI on CD123^+^ DCs facilitate antigen internalization, presentation and Th2 stimulation remains to be examined. Based on the tolerogenic function of CD123^+^ DCs [27–29], studies on the functional outcome of FcεRI-mediated allergen uptake by CD123^+^ DCs are warranted. Of note, we have previously demonstrated that pDCs are unable to stimulate grass-allergen specific Th2 responses in contrast to CD1c^+^ DCs [6]. Although no allergen-specific Abs were added, these DCs were purified from allergic donors and are thus likely to have IgE bound to FcεRI and IgE-mediated uptake can therefore have been one of the mechanisms by which the CD1c^+^ DCs internalized and presented the allergen. Thus, the report supports the idea that pDCs and mDCs process allergen differently. To get a more detailed view of the process, a similar experiment could be performed with the addition of allergen-specific IgE and the IgE uptake in pDCs as well as mDCs upon allergen stimulation could be monitored by fluorescence microscopy. Also, the effect of IgE-stimulation of pDC and mDCs, respectively, on subsequent T-cell responses could be examined. The majority of the CLRs assessed in this study were significantly down regulated by AIT, with the most pronounced effects observed on CD141^+^ and CD1c^+^ DCs. By contrast, no changes were detected on CD16^+^ DCs, and the two CLRs that were altered on CD123^+^ DCs showed very low expression, making it difficult to speculate on their biological influences. The significant reduction in CD206 expression on CD141^+^ and CD1c^+^ DCs, and of Dectin-2 on CD141^+^ DCs, after 1 year of AIT, may be indicative of modified cellular events, possibly tolerogenic, in response to allergen recognition, as these CLRs have been shown to interact with various allergen components and mediate Th2 responses [9,11,12]. Additionally, CD209 can interact with allergens [10,13] and after 1 year of AIT, expression of CD209 on CD141^+^ DCs was reduced by all donors (S4 Fig, p = 0.0651). Importantly, altered ability of CD1c^+^ and CD141^+^ DCs to interact with allergens could potentially affect the allergic response as functional assessments have shown that these subsets are able to stimulate Th2 responses [6,33]. In line with our data, expression of another pattern recognition receptor, Toll-like receptor (TLR) 2, has been demonstrated to be reduced on CD11c^+^ mDCs by AIT for venom allergy [4]. Expression of Dectin-1 was significantly decreased on CD1c^+^ DCs during AIT, which may contribute to the beneficial effect of AIT as this receptor is shown to be involved in allergic responses [14]. On the other hand, a recent publication showed that Dectin-1–activated mDCs decreased Th2 responses, whereas Dectin-1-activated pDCs promoted Th2 responses [16]. However, it was not clarified whether the decreased Th2 response upon mDC activation via Dectin-1 is mediated by CD1c^+^, CD141^+^ and/or CD16^+^ mDCs. Thus, the decrease in Dectin-1 on CD1c^+^ DCs shown in the current study could have a beneficial or a disadvantageous effect on the allergic reaction [16]. In addition to the CLRs presently known to be associated with allergic responses, this study showed that expression of CD280 on CD141^+^ DCs was clearly decreased upon AIT. Allergen-recognition by CLRs has only recently become known [12] and whether CD280 interact with allergens remains to be explored.

In contrast to the aforementioned down regulated CLRs, a marked (four-fold) and statistically significant up regulation of DNGR1 expression was demonstrated on CD141^+^ DCs after 8 weeks as well as 1 year of AIT. DNGR1 is uniquely present on CD141^+^ DCs and we have recently shown that the frequency of DNGR1 positive cells is lower on CD141^+^ DCs from allergic as compared to non-allergic donors [20]. Notably, the present AIT patients displayed a restored DNGR1 expression similar to that of cells from non-allergic donors. In line with our data, AIT has previously been shown to eliminate differences between allergic and non-allergic subjects regarding the phenotypes of DC [34]. Specifically, Wang *et al*. demonstrated that expression of TLR4 and CD86 by MoDCs after stimulation with house dust mite allergen and LPS, respectively, more closely resembled cells from non-allergic individuals after AIT, as compared to before treatment [34]. DNGR1-targeted antigen delivery has been shown to induce CD8^+^ T-cells [21] and murine studies have shown that CD8^+^ T-cells are critical for the suppressive phenotype of regulatory T-cells in allergic disease [22]. Thus, the restored DNGR1 expression by AIT supports a potential role of this CLR in the development of tolerance. In support of this, it has been shown that targeting DNGR1 in absence of adjuvant give rise to a regulatory T-cell response in mice [19]. To get further insights into the relevance of DNGR1 in tolerance, one can monitor DNGR1 expression in parallel with presence of regulatory T-cells during AIT, to find out if these parameters correlate.

In summary, the present data advances current knowledge on DC subset frequencies and particularly alterations by AIT of FcR and CLR expression profiles of DC subsets. Specifically, the study demonstrates the following AIT-induced changes: 1) up regulation of the CD123^+^ DCs subset, which is potentially relevant for induction of tolerance, 2) modification of FcR expression by CD123^+^ and CD141^+^ DCs, suggesting an adjusted Ig-mediated allergen internalization 3) altered CLR expression preferentially by CD141^+^ and CD1c^+^ DCs, suggesting modified allergen recognition and allergen presentation to T-cells and 4) restored expression of DNGR1 on CD141^+^ DCs, suggesting a role in the development of tolerance. Taken together, these observations describe cellular events possibly linked to a mechanistic role of DC subsets in development of allergen tolerance and may offer new targets for modulation of immune responses.

## Supporting Information

S1 Fig Serum levels of allergen-specific IgG4 and IgE in AIT subjects. Levels of IgG4 and IgE specific for the allergens used for treatment (see Table I) were measured in serum from t0, t8w and t1y (serum at t0 missing for donor 5). The different colors refer to the individual donors and shapes to allergens. D-donor; Art v-*Artemisia vulgaris*; Bet v-*Betula verrucosa*; Der p-*Dermatophagoides pteronyssinus*; Fel d-*Felis domesticus*; Phl p-*Phleum pratense*; t-time point; w-weeks; y-year.
(TIF)

S2 Fig The expression of FcRs and CLRs was analyzed by comparing the sample stained with anti-FcR/CLR Ab with the FMO control sample (stained identically but without anti-FcR/CLR Ab). One representative donor stained for CD64 expression at t0 and t1y is shown. All CLR/FcR samples were analyzed in the same manner for all time points. Net percent positive cells were subsequently calculated for each DC subset as percent positive for sample minus percent positive for the FMO control.
(TIF)

S3 Fig Expression of CD32 by CD141^+^ DCs. Flow cytometric analysis performed at t0 (before AIT) and t8w (≅8 weeks of AIT) and statistical analysis performed using Friedman’s test followed by Dunn's multiple comparison test.
(TIF)

S4 Fig Expression of CD209 by CD141^+^ DCs. Flow cytometric analysis performed at t0 (before AIT) and t1y (≅1 year of AIT) and statistical analysis performed using Friedman’s test followed by Dunn's multiple comparison test.
(TIF)

S1 Table Number of matched AIT donors analyzed by Friedman test. (DOCX)

## Abbreviations

AIT: Allergen specific immunotherapy
CLEC: C-type lectin domain family
CLECSF: C-type lectin superfamily member
CLR: C-type lectin receptors
DC: Dendritic cell
DCIR: Dendritic cell immunoreceptor
DC-SIGN: Dendritic cell-specific ICAM-3-grabbing non-integrin 1
Dectin: Dendritic cell-associated C-type lectin
DNGR1: Dendritic cell natural killer lectin group receptor 1
FcR: Fc receptor
FMO: Fluorescence minus one
Ig: Immunoglobulin
mDC: Myeloid dendritic cell
MR: Macrophage mannose receptor 1
MRC2: C-type mannose receptor 2
MoDC: Monocyte derived dendritic cells
PCA: Principal Component Analysis
pDC: Plasmacytoid dendritic cell
TLR: Toll-like receptor
