# Supplementary figures and images for: Allergen-Specific Immunotherapy Alters the Frequency, as well as the FcR and CLR Expression Profiles of Human Dendritic Cell Subsets
Source: PLoS One. 2016 Feb 10;11(2):e0148838. doi: 10.1371/journal.pone.0148838 (PMC4749279; doi:10.1371/journal.pone.0148838)

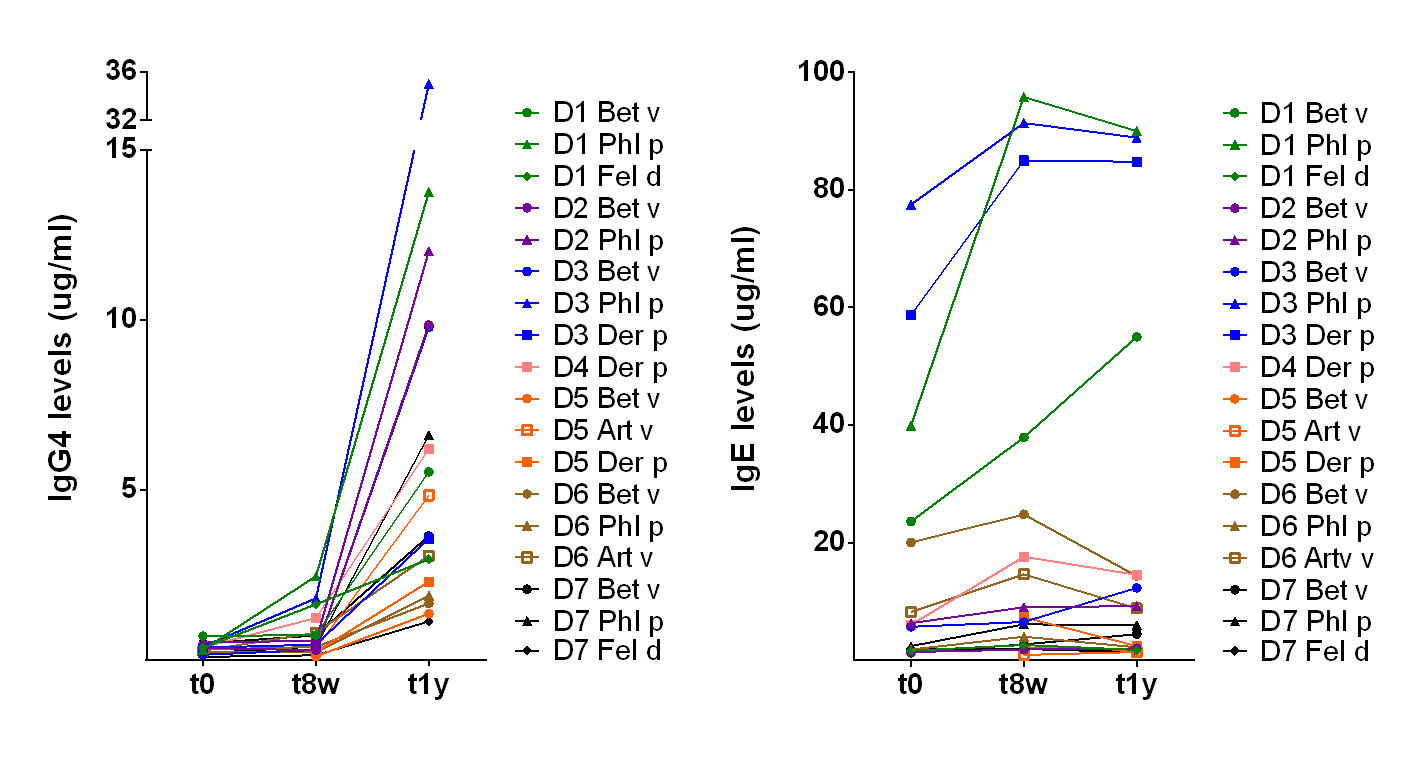

Supplement: S1 Fig — Levels of IgG4 and IgE specific for the allergens used for treatment (see Table I) were measured in serum from t0, t8w and t1y (serum at t0 missing for donor 5). The different colors refer to the individual donors and shapes to allergens. D-donor; Art v-Artemisia vulgaris; Bet v-Betula verrucosa; Der p-Dermatophagoides pteronyssinus; Fel d-Felis domesticus; Phl p-Phleum pratense; t-time point; w-weeks; y-year. (TIF) [file pone.0148838.s001.tif]

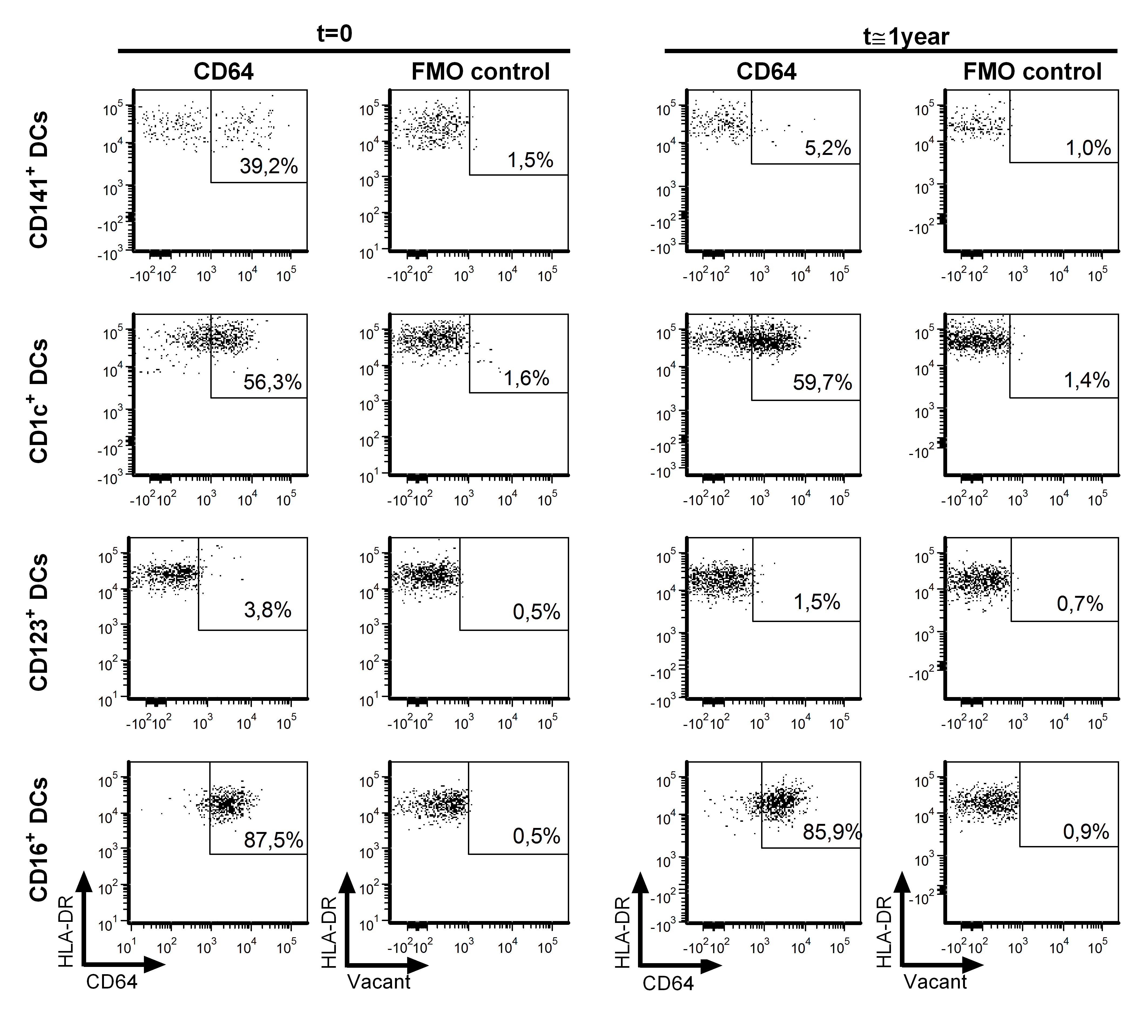

Supplement: S2 Fig — One representative donor stained for CD64 expression at t0 and t1y is shown. All CLR/FcR samples were analyzed in the same manner for all time points. Net percent positive cells were subsequently calculated for each DC subset as percent positive for sample minus percent positive for the FMO control. (TIF) [file pone.0148838.s002.tif]

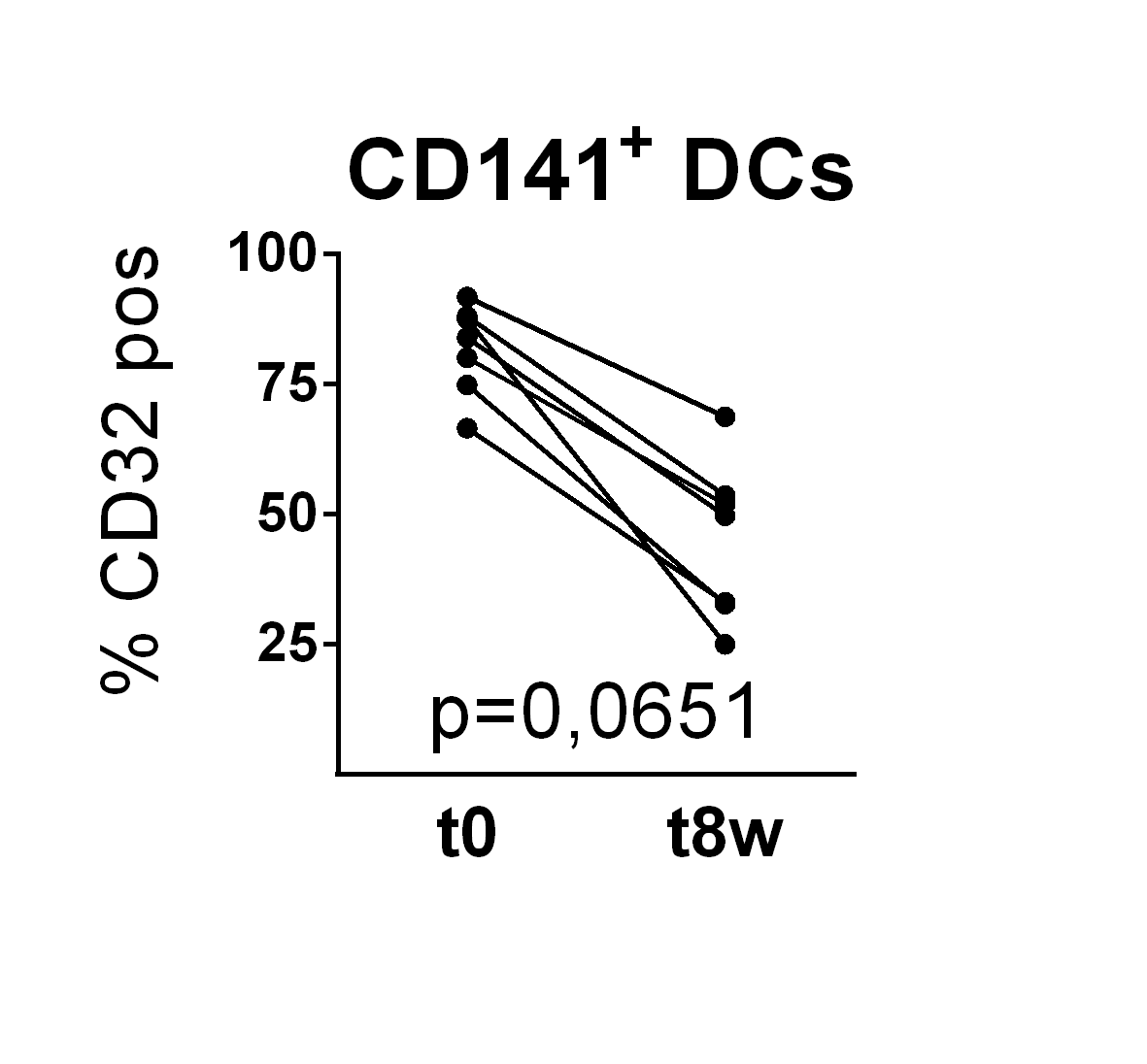

Supplement: S3 Fig — Flow cytometric analysis performed at t0 (before AIT) and t8w (≅8 weeks of AIT) and statistical analysis performed using Friedman’s test followed by Dunn's multiple comparison test. (TIF) [file pone.0148838.s003.tif]

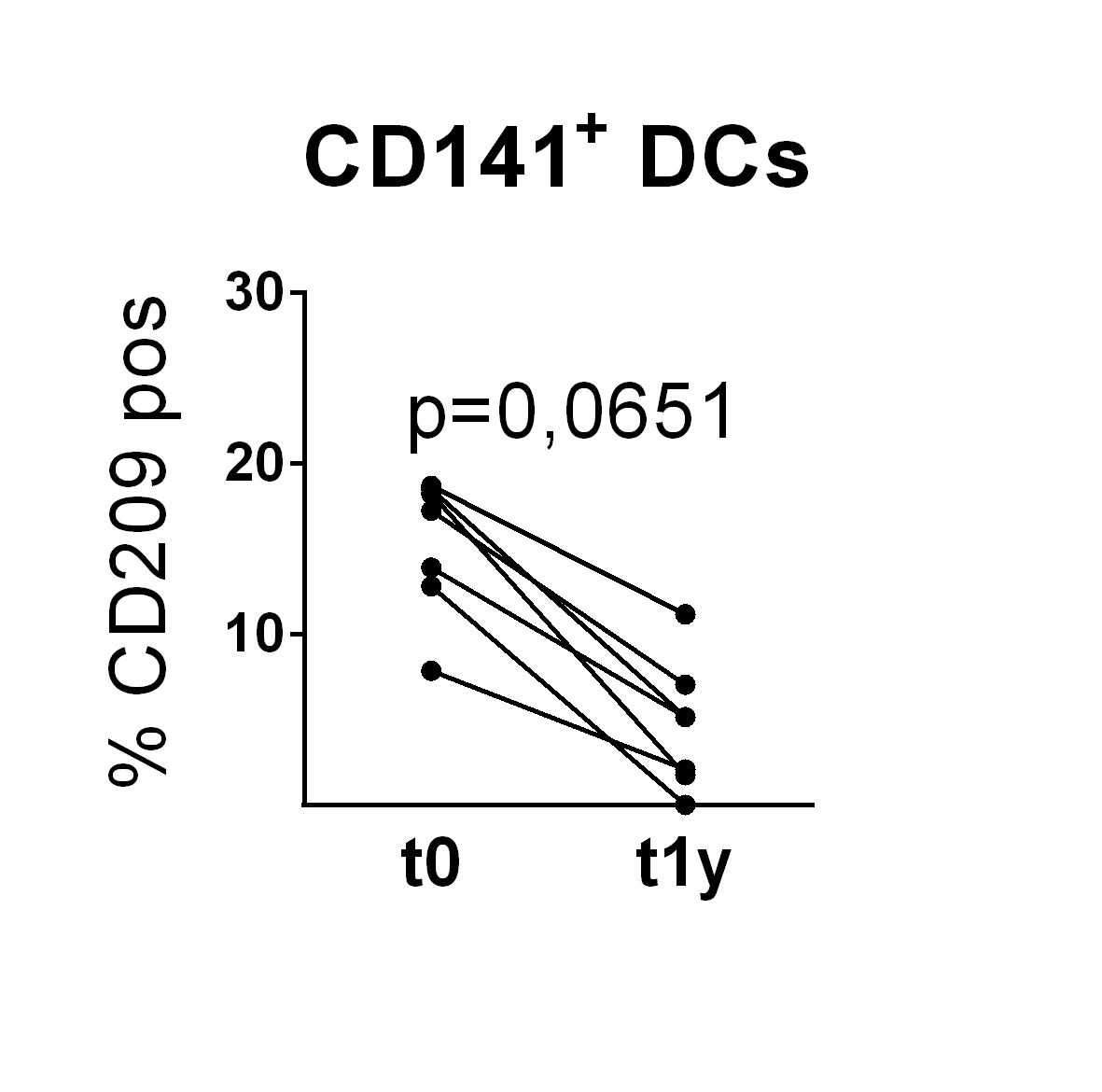

Supplement: S4 Fig — Flow cytometric analysis performed at t0 (before AIT) and t1y (≅1 year of AIT) and statistical analysis performed using Friedman’s test followed by Dunn's multiple comparison test. (TIF) [file pone.0148838.s004.tif]
