# Supplementary material for: Allergen-Specific Immunotherapy Alters the Frequency, as well as the FcR and CLR Expression Profiles of Human Dendritic Cell Subsets
Source: PLoS One. 2016 Feb 10;11(2):e0148838. doi: 10.1371/journal.pone.0148838 (PMC4749279; doi:10.1371/journal.pone.0148838)
Supplement: S1 Table — (DOCX) [file pone.0148838.s005.docx]

**Supporting table**

**S1 Table. Number of matched AIT donors analyzed by Friedman test.**

|  | **CD141^+^ DCs** | **CD1c^+^ DCs** | **CD123^+^ DCs** | **CD16^+^ DCs** |
| --- | --- | --- | --- | --- |
| **CD209/DC-SIGN** | 7 | 7 | 7 | 7 |
| **CD301/CLECSF14** | 7 | 6 | 7 | 6 |
| **CD206/MR** | 7 | 7 | 7 | 7 |
| **CD207/Langerin** | 7 | 7 | 6 | 6 |
| **CD205/DEC205** | 7 | 7 | 7 | 7 |
| **DCIR/CLECSF6** | 7 | 7 | 7 | 7 |
| **DNGR1/CLEC9A** | 5 | 5 | 5 | 5 |
| **CD280/MRC2** | 7 | 7 | 7 | 7 |
| **Dectin-2/CLEC6A** | 5 | 4 | 5 | 5 |
| **Dectin-1/CLEC7A** | 5 | 5 | 5 | 5 |
